# A Deletion in the N-Myc Downstream Regulated Gene 1 (*NDRG1*) Gene in Greyhounds with Polyneuropathy

**DOI:** 10.1371/journal.pone.0011258

**Published:** 2010-06-22

**Authors:** Cord Drögemüller, Doreen Becker, Barbara Kessler, Elisabeth Kemter, Jens Tetens, Konrad Jurina, Karin Hultin Jäderlund, Annette Flagstad, Michele Perloski, Kerstin Lindblad-Toh, Kaspar Matiasek

**Affiliations:** 1 Institute of Genetics, Vetsuisse Faculty, University of Berne, Berne, Switzerland; 2 Chair for Molecular Animal Breeding and Biotechnology, Laboratory for Functional Genome Analysis, Gene Center, Ludwig-Maximilians-University Munich, Munich, Germany; 3 Institute for Animal Breeding and Husbandry, Christian-Albrechts-University Kiel, Kiel, Germany; 4 Tierklinik Haar, Haar, Germany; 5 Department of Clinical Sciences, Swedish University of Agricultural Sciences, Uppsala, Sweden; 6 Department of Companion Animal Clinical Sciences, Norwegian School of Veterinary Science, Oslo, Norway; 7 Department of Clinical Sciences, Faculty of Life Sciences, University of Copenhagen, Copenhagen, Denmark; 8 Broad Institute of Harvard and MIT, Cambridge, Massachusetts, United States of America; 9 Department of Medical Biochemistry and Microbiology, Uppsala University, Uppsala, Sweden; 10 Neuropathology Laboratory, Animal Health Trust, Newmarket, United Kingdom; University of Florida, United States of America

## Abstract

The polyneuropathy of juvenile Greyhound show dogs shows clinical similarities to the genetically heterogeneous Charcot-Marie-Tooth (CMT) disease in humans. The pedigrees containing affected dogs suggest monogenic autosomal recessive inheritance and all affected dogs trace back to a single male. Here, we studied the neuropathology of this disease and identified a candidate causative mutation. Peripheral nerve biopsies from affected dogs were examined using semi-thin histology, nerve fibre teasing and electron microscopy. A severe chronic progressive mixed polyneuropathy was observed. Seven affected and 17 related control dogs were genotyped on the 50k canine SNP chip. This allowed us to localize the causative mutation to a 19.5 Mb interval on chromosome 13 by homozygosity mapping. The *NDRG1* gene is located within this interval and *NDRG1* mutations have been shown to cause hereditary motor and sensory neuropathy-Lom in humans (CMT4D). Therefore, we considered *NDRG1* a positional and functional candidate gene and performed mutation analysis in affected and control Greyhounds. A 10 bp deletion in canine *NDRG1* exon 15 (c.1080_1089delTCGCCTGGAC) was perfectly associated with the polyneuropathy phenotype of Greyhound show dogs. The deletion causes a frame shift (p.Arg361SerfsX60) which alters several amino acids before a stop codon is encountered. A reduced level of *NDRG1* transcript could be detected by RT-PCR. Western blot analysis demonstrated an absence of NDRG1 protein in peripheral nerve biopsy of an affected Greyhound. We thus have identified a candidate causative mutation for polyneuropathy in Greyhounds and identified the first genetically characterized canine CMT model which offers an opportunity to gain further insights into the pathobiology and therapy of human *NDRG1* associated CMT disease. Selection against this mutation can now be used to eliminate polyneuropathy from Greyhound show dogs.

## Introduction

Inherited neuropathies comprise all neurological phenotypes arising from genetic defects that lead to abnormalities of important peripheral nerve proteins, or impair multisystemic metabolic pathways [1]. Several subtypes of inherited polyneuropathies were delineated and classified as hereditary motor and sensory neuropathies (HMSN), hereditary motor neuropathies (HMN), and hereditary sensory (and autonomic) neuropathies (HSAN) [2]. These clinically heterogeneous phenotypes affecting the peripheral nerves are grouped together as Charcot-Marie-Tooth (CMT) disease. According to nerve conduction studies and/or biopsy examination, CMT phenotypes are classified into the most prevalent ‘demyelinating’, the rare ‘axonal’ or ‘neuronal’, and several ‘mixed’ forms. CMT condition affects an estimated 8 to 41 per 100,000 people worldwide [3]. More than 40 genes with distinct mutations have been described and most of them lead to autosomal dominant forms of CMT [4]. Recessive mutations are less frequent, and many rare CMT forms still await genetic clarification. Currently, there is no drug therapy for human CMT disease available. Therefore, studying suitable defined animal models may be useful for the identification of therapeutic targets and approaches [5].

Domestic animals constitute an essential complement to rodent models and are an underutilized resource in biological research and as a model for human diseases [6]. CMT diseases also occur in dogs [7] and the dog may represent a better model for human CMT than genetically engineered mice because of its larger body size, relatively long life expectancy, and the resulting similarities to humans. Specific CMT diseases have been described in several canine breeds including Great Dane [8], Rottweiler [9], Dalmatian [10], Alaskan Malamute [11], Leonberger [12], German Shepherd [7], Italian Spinoni [13], Bouvier des Flandres [14], Border Collie [15], [16], Pyrenean Mountain dog [17], and Miniature Schnauzer [18]. The underlying genetic defect has not yet been elucidated for any of the canine CMT forms.

We have observed a severely progressive mixed form of polyneuropathy, with juvenile onset, in a pedigree of closely related Greyhound show dogs. We investigated peripheral nerve biopsies of affected dogs for phenotypic characterization of this new canine CMT disease. Subsequently, we employed a positional cloning approach to identify the most likely cause for polyneuropathy in Greyhound show dogs.

## Results

### Clinical characterization of polyneuropathy

**Figure 1:**
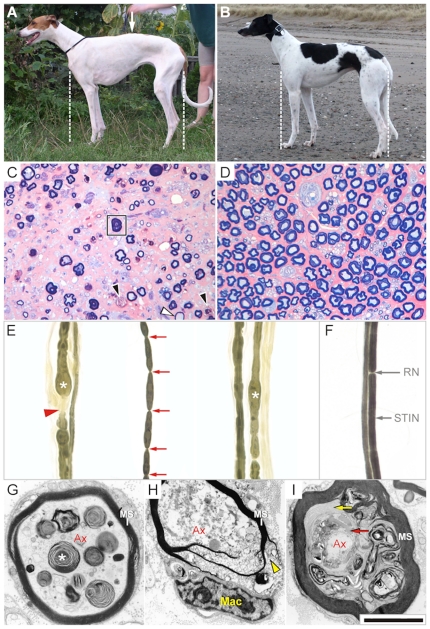
Polyneuropathy in Greyhound show dogs. Most consistent findings in affected dogs (**A**, **C**, **E**, **G**, **H**, **I**) compared to healthy controls (**B**, **D**, **F**). (Scale bar: 100 µm in C and D; 125 µm in E and F; 10 µm in G and H; 17 µm in I). (**A**, **B**) General appearance: Affected dogs during ambulatory stages present with abnormal posture characterized by narrow limb positioning and mildly kyphotic backline (arrow). (**C**, **D**) Light microscopic nerve findings: At advanced disease stages mixed nerves show a drop out of large myelinated A(alpha) fibres, including motor fibres and Ia/b afferents. Remaining fibres frequently present with axonal atrophy and subsequent myelin sheath adjustment (frame). Multiple myelin ovoids indicate Wallerian like degeneration (black arrowheads). Hypomyelinated profiles are occasionally seen (white arrowhead). (**E**, **F**) Teased nerve fibres: Nerve of affected dogs show multifocal paranodal demyelination (red arrowhead), frequently associated with axonal swelling (asterisk). Numerous fibres with and without other abnormalities are affected by myelin sheath segmentation (red arrows), consistent with early stage I of Wallerian degeneration. Compare to normal myelinated nerve fibres with discernible Ranvier's nodes (RN) and stereotypic internodal segments (STIN). (**G**) Electron microscopy: Swollen axons (Ax) show an accumulation of dysmorphic organella, electron dense granules, and lamellated bodies (asterisk). In spite of increased axon diameters the myelin sheath (MS) most often is disproportionately thin. (**H**) Electron microscopy: Actual fibre degeneration occasionally starts with axoplasmic disintegration (Ax) and macrophageal (Mac) myelin stripping from the abaxonal Schwann cell cytoplasm extending along the major dense lines (yellow arrowhead). (**I**) Electron microscopy: Fibres with shrunken axons (Ax) show adaxonal myelin sheath protrusion, dyscompaction of the inner myelin layers (yellow arrow), and accumulation of dysmorphic mitochondria and curvilinear bodies (red arrow).

The polyneuropathy of juvenile Greyhound show dogs becomes clinically apparent between three to nine months of age. Owners of affected dogs reported exercise intolerance and walking difficulties such as high stepping gait and bunny hopping in the early stages of the disease (Video S1). In the later stages, the disease was characterized by severe muscle atrophy, ataxia and dysphonia (Video S2). No behavioral abnormalities, retardation or learning difficulties were observed. On neurological examination, all dogs were alert, bright, and responsive. Neurological signs of affected dogs were progressive ataxia and tetraparesis, delayed proprioceptive placing reactions, hyporeflexia, distal limb muscle atrophy, and inspiratory stridor (Figure 1A). Depending on the stage of disease, the gait was moderately to markedly ataxic on all four limbs and appeared short-strided. Later, however, elbows and stifles were abducted and the carpus was hyperextended, while elbow, hip, stifle, and tarsal joints appeared flexed. Trembling and collapse occurred after exercise. Muscle tone was moderately to severely reduced, with lack of resistance to passive joint movement in either direction. Postural reactions seemed unaffected in the early stages. With disease progression, proprioceptive deficits and laryngeal involvement occurred. Most strikingly, myotatic reflexes were reduced or absent. One affected dog had decreased withdrawal in all four limbs. The cutaneous trunci reflex was bilaterally absent in another case. Cranial nerve function did not seem to be affected until the terminal stages of disease. Another Greyhound developed marked paresis of the intercostal muscles and diaphragm, leading to respiratory distress. On electromyography, a pattern of multifocal spontaneous discharges was recorded from appendicular muscles in some dogs. Fibrillation potentials were most common, followed by positive sharp waves (Figure S1A–C). In particular, the interosseous, cranial tibial, and gastrocnemius muscles were affected. Motor and sensory nerve conduction velocities were reduced (Figure S1D). Repetitive nerve stimulation was unremarkable and compound muscle action potential amplitude was decreased.

### Pathological nerve and muscle findings

All nerve specimens revealed a mild (up to 25%) to marked (25–50%) reduction in myelinated nerve fibre density associated with variable endoneurial fibrosis and subperineurial oedema (Figure 1C). More than 25% of remaining fibres showed marked to severe loss of circularity due to para- and internodal crenation, as well as due to outfolded myelin loops at paranodes. The mean axonal calibre appeared reduced albeit diameter distribution presented a large scatter of values due to multiple axonal enlargements in more than 10% of large myelinated teased fibres at both stereotypic internodal segments (STIN) and paranodes (Figure 1E and F). Compared to intact axons, these varicosities stained hyperazurophilic and granular. Electron microscopically, axonal swellings consisted of axoplasmic dense bodies, distorted mitochondria, paracrystalline bundles and concentric lamellar bodies (Figure 1G). Adjacent to these segments adaxonal Schwann cell compartments contained abundant granulofilamentous inclusions (Figure 1H). Longitudinal sections uncovered multiple outpouchings of axoplasm extruding into neighbouring uncompacted myelin of inter- and paranodes. Moreover, mild to marked hyperplasia of the axon-Schwann cell-network was identified in about 10% of large myelinated fibres, extending deeply into STINs. Dilation of Schmidt-Lanterman incisures was a frequent finding and many large myelinated A (alpha) fibres showed ovoid formation consistent with Wallerian degeneration stage I to III (Figure 1C and E). In about 5% of fibres with different types of pathology, there were multiple widened nodes of Ranvier and a paranodal demyelination was noted in fibres with and without colocalizing paranodal axon swelling. In rare regenerating fibres, heminodes and intercalating internodes were seen in clusters. Severe axonal atrophy went along with paranodal myelin sheath instability indicated by redundant loops and tomacula (Figure 1I).

Variable degrees of neurogenic atrophy were present within skeletal muscle samples, consistent with chronic-active denervation. Atrophy ranged from singular fibres and small fibre groups, with occasional necrosis, undergoing myophagocytosis to large groups with scattered, pyknotic nuclear clumps. Other changes indicating chronic denervation included perimysial lipid accumulation and loss of myelinated nerve fibres within intramuscular nerve branches.

### Genome-wide homozygosity mapping of the polyneuropathy mutation

**Figure 2:**
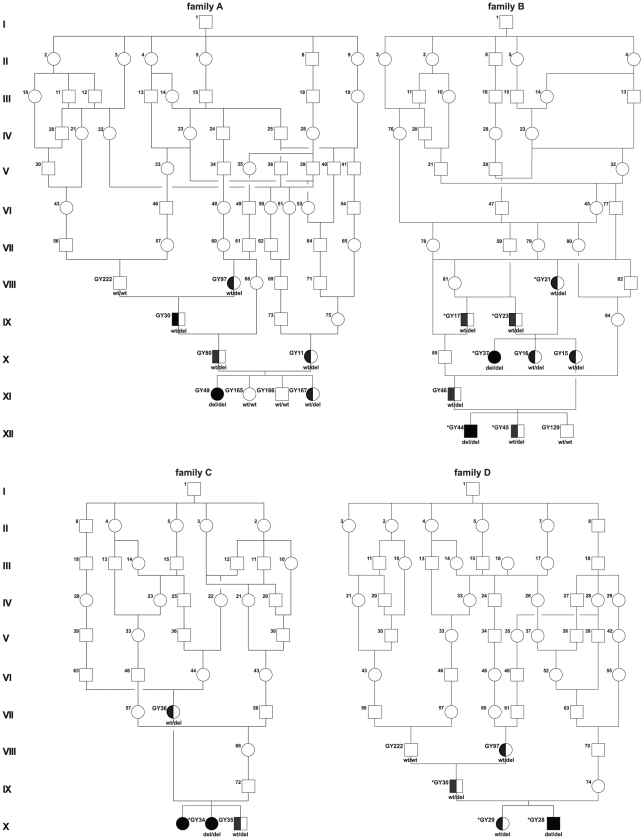
Pedigrees of sampled Greyhound show dogs with polyneuropathy. DNA samples were available only from GY numbered dogs. Animals genotyped on the SNP chip are indicated by asterisk. The genotypes for the *NDRG1* exon 15 deletion are given below the symbols. Due to the very close relationships four single pedigrees are shown, where some individuals indicated with identical numbers are represented in multiple pedigrees (also in Figure 3).

**Figure 3:**
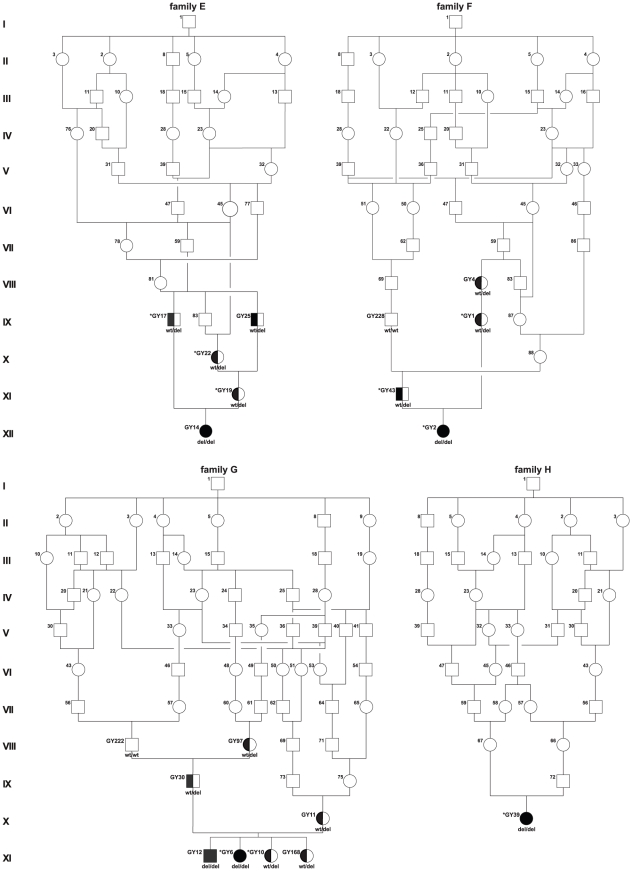
Pedigrees of sampled Greyhound show dogs with polyneuropathy. DNA samples were available only from GY numbered dogs. Animals genotyped on the SNP chip are indicated by asterisk. The genotypes for the *NDRG1* exon 15 deletion are given below the symbols. Due to the very close relationships four single pedigrees are shown, where some individuals indicated with identical numbers are represented in multiple pedigrees (also in Figure 2).

**Figure 4:**
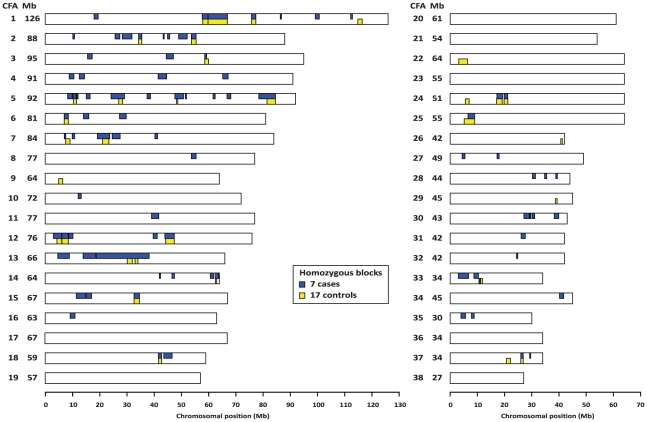
Genome-wide homozygosity mapping of the polyneuropathy mutation. After genotyping approximately 50,000 fairly uniformly distributed SNP markers homozygous blocks were identified independently across 7 affected and 17 control dogs. Note that only on canine chromosome (CFA) 13 a unique large homozygous block was identified in the cases.

We collected blood from dogs affected by polyneuropathy sampling from the international Greyhound show dog population. The parents of the 11 affected dogs (8 males, 3 females) were all healthy. The pedigrees of the 11 affected dogs show many inbreeding loops (Figure 2 and 3). While the many complex loops make it difficult to be absolutely certain, the breeding history was still consistent with a monogenic autosomal recessive inheritance of polyneuropathy. Remarkably, all affected dogs were related to a single popular sire born in 1968 on both the maternal and paternal sides of their respective pedigrees. Under this scenario the polyneuropathy affected dogs were expected to be identical by descent (IBD) for the causative mutation and flanking chromosomal segments. Therefore, we decided to apply a homozygosity mapping approach to determine the position of the mutation in the canine genome. We genotyped approximately 50,000 evenly spaced SNPs in 7 cases and 17 related and unrelated control dogs. We analyzed the 7 genotyped cases for extended regions of homozygosity with simultaneous allele sharing. A total of 83 genomic regions larger than 300 kb fulfilled our search criteria (Figure 4). The size of homozygous blocks ranged between 0.3 and 19.5 Mb with a mean size of about 2 Mb and a median of 1.5 Mb. Analyzing the 17 genotyped controls revealed 33 blocks of homozygosity between 0.3 and 7.1 Mb size (Figure 4). Only on CFA 13, in a region containing 538 SNP markers corresponding to a 19.5 Mb interval from 18.7–38.2 Mb (Figure 4), were all 7 affected genotyped dogs were homozygous, but the controls were not homozygous.

### Identification and characterization of a functional candidate gene

**Figure 5:**
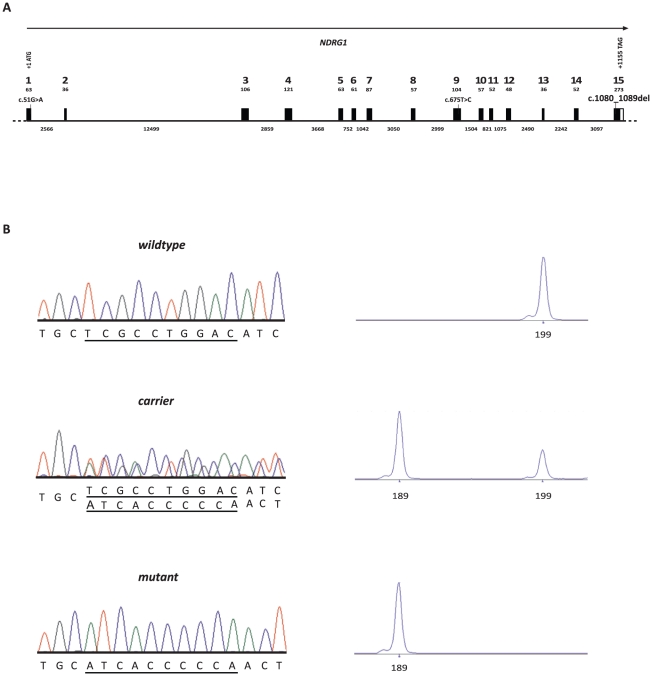
*NDRG1* mutation analysis. (**A**) Canine *NDRG1* gene structure. (**B**) Electropherograms of the *NDRG1* c.1080_1089delTCGCCTGGAC mutation. Representative sequence traces of PCR products amplified from genomic DNA of 3 dogs with the different genotypes are shown. The presence of the mutation can be directly visualized by fragment size analysis of a fluorescently labeled PCR product containing the mutation. Note that the mutant allele with the 10 bp-deletion is present in homozygous form only in polyneuropathy affected dogs.

As the quality of the canine genome annotation is not yet perfect, we inferred the gene annotation of the mapped interval from the corresponding human interval. The canine polyneuropathy interval on CFA 13 corresponds to a segment from 117.2–141.3 Mb on HSA 8q24. This human interval contains 123 annotated genes including 14 annotated pseudogenes (NCBI MapViewer, build 37.1). A careful inspection of these genes and database searches of their presumed function revealed *NDRG1* as a functional candidate gene within the critical interval at 134.3 Mb on HSA 8 and 32.7 Mb on CFA 13, respectively. We performed a mutation analysis in 2 affected dogs, 2 obligate carriers and 2 control dogs. This analysis revealed ten polymorphisms in comparison to the dog reference genome sequence, including 9 SNPs and a single 10 bp deletion (Table S1). Of these polymorphisms only the deletion within exon 15 (c.1080_1089delTCGCCTGGAC, Figure 5) showed perfect association to the polyneuropathy phenotype. We genotyped additional dogs for the mutation and found perfect concordance between the presence of the deletion and the polyneuropathy phenotype in all available affected dogs, while the deletion was present in the heterozygous state in all obligate carriers (Figure 2 and 3; Table 1). Four grandmothers, a single grandfather, and 8 out of 11 healthy full-sibs of affected dogs were also heterozygous. None of 245 unrelated healthy Greyhound show dogs had the homozygous del/del genotype, but 62 (∼25%) were also carriers. The mutant allele was absent from 112 Greyhound racing dogs, and was also not present in any of the 91 control dogs from different breeds including 7 other types of sight hounds (Table 1).

**Table 1:** Association of the *NDRG1* mutation with the polyneuropathy phenotype.

Breed	free	carrier*	affected	total
Greyhound (show)	189	84	10	283
Greyhound (racing)	112			112
Afghan Hound	2			2
Borzoi	1			1
Galgo Español	1			1
Italian Greyhound	1			1
Irish Wolfhound	1			1
Saluki	1			1
Whippet	9			9
other breeds (n = 75)	75			75

*Parents of affected offspring were classified as obligate carriers (n = 12).

### Analysis of *NDRG1* expression in peripheral nerve tissue

**Figure 6:**
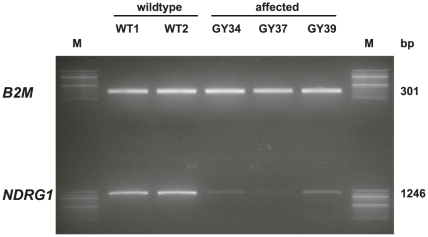
RT-PCR of *NDRG1* expression in peripheral nerve biopsies. A strong band of the expected size of wild-type *NDRG1* transcript was amplified from two 2 control dogs. Three polyneuropathy affected Greyhound show dogs yielded clearly weaker band intensities. In comparison, the detected beta 2-microglobulin gene expression was quite similar between all samples. (M: 100 bp DNA ladder).

**Figure 7:**
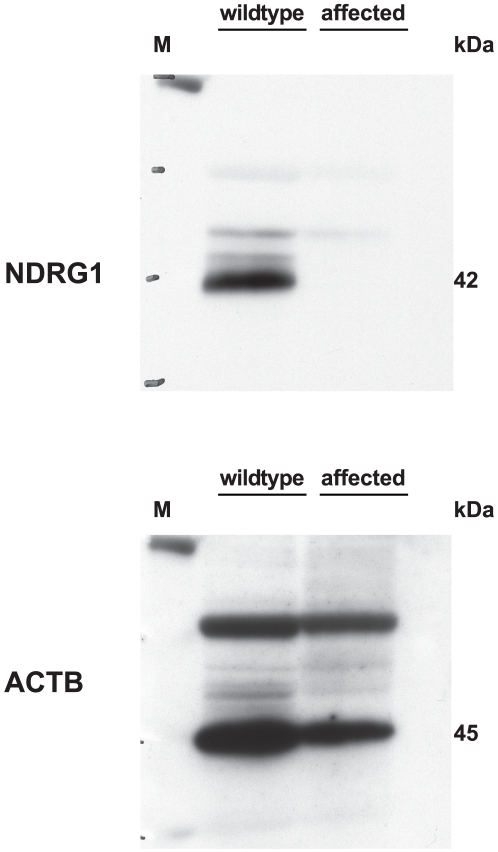
Western Blot analysis of NDRG1 protein expression in peripheral nerve biopsies. A strong band of the expected size of wild-type NDRG1 protein (41.7 kDa) was detected in the protein sample from the healthy control dog. Protein extract from the nerve of a polyneuropathy affected dog yielded no specific band. In comparison, a band of the expected size of beta-actin (ACTB, 45 kDa) was detected in both samples.

RT-PCR on peripheral nerve cDNA confirmed that the *NDRG1* mRNA is normally spliced in Greyhounds with polyneuropathy. We sequenced the cDNA from 3 affected dogs and it contained the 10 bp deletion in a homozygous state confirming that the mutant mRNA is expressed. Semi-quantitative RT-PCR revealed evidence that the expression level of the mutant *NDRG1* RNA transcript is considerably reduced in comparison to wild-type control dogs (Figure 6). The deletion is predicted to result in a frameshift beginning with amino acid residue 361. Notably, the mutation does not lead to premature termination of translation but predicts a C-terminal mutated protein (p.Arg361SerfsX60) that is longer than the wild-type protein (384 amino acid residues). To evaluate the functional consequences of this deletion we performed a western blot on protein extracts from neural tissue of an affected dog and a healthy control dog to investigate whether the predicted longer mutant NDRG1 protein is expressed. The western blot of the control dog showed a strong band of the expected size for the NDRG1 protein. However, in contrast to our initial hypothesis, no NDRG1 specific band was detected in neural tissue of the homozygous mutant affected Greyhound show dog (Figure 7). Therefore the transcript was present, but appeared not to be translated to protein.

## Discussion

Most known neuropathies in dogs manifest in adolescence or adulthood [7]. However, the juvenile polyneuropathy in Greyhounds stands out by an early onset of about three to nine months of age. After clinical signs appeared none of the animals survived longer than ten months. If not euthanized earlier on request of the owners, progression of laryngeal paresis/paralysis and phrenic neuropathy is going to inflict lethal consequences. All dogs of this study suffered from a polyneuropathy that could be classified as mixed or predominantly axonal disease based on evidence of axonal dystrophy from electron microscopy.

The Greyhound is a breed of hunting dogs that has been bred primarily for chasing game. Nowadays, the vast majority of Greyhounds are bred for professional racing, but partially also as pedigree show dogs and family pets. Although they officially belong to a single breed, breeding lines of show and racing Greyhounds have been almost completely separated for 60 years. It seems likely that the deleterious mutation arose in the show dog population, as no carrier of the *NDRG1* mutation has been identified among the racing dog variety. The fact, that about a quarter of all genotyped Greyhound show dogs carries the mutation for polyneuropathy, might be explained by an intensive use of particular male carriers selected for desired show traits during the establishment and/or expansion of the Greyhound show dog population. The founding mutation presumably happened at least nine to eleven generations before the reported cases occurred (Figure 2). As shown for the affected dogs, also all genotyped carriers of the *NDRG1* mutation can be traced back to the popular sire born in 1968. If this popular Greyhound show sire disseminated the deleterious mutation, subsequent inbreeding among the descendants increased the likelihood of polyneuropathy emerging. The observed high allele frequency (∼25%) of the polyneuropathy mutation together with the high number of homozygous SNPs (∼60%) in the genotyped dogs including affected dogs and controls, suggest a high level of inbreeding within the Greyhound show dog population. This confirms the general observation, that the population structure of modern dogs makes them particularly useful for positional cloning studies, as a single breed may resemble recombinant inbred lines [19]. This reduced genetic diversity within dog breeds leads to large haplotype blocks, as detected in the examined Greyhound dogs in this study.

The aim of this study was to identify the gene responsible for polyneuropathy of Greyhound show dogs. Homozygosity mapping identified a large segment on CFA 13. In comparison to a recent study, where the causative gene for recessively inherited osteogenesis imperfecta in Dachshunds was mapped to a unique homozygous chromosome segment of 5.8 Mb after genotyping of only 5 affected dogs on the 50k canine SNP chip [20], we here identified several extended Mb regions of homozygosity in the 7 affected dogs including the longest of 19 Mb containing the mutation. The long region surrounding the mutation suggests that the polyneuropathy mutation is quite young, whereas the high number of homozygous regions in both cases and controls suggests a generally inbred population. The fact that healthy controls shared some of these homozygous segments and had identical alleles helped us to focus on the chromosome segment of 19.5 Mb on CFA 13. We note that the mapping of older mutations may require many more affected dogs, because the associated IBD haplotype is usually much smaller, due to independent recombination events over several generations [19]. Still, this study shows once again, that the use of genome-wide canine SNP data enables very efficient positional cloning projects of Mendelian traits, even if only very few samples are available.

The mapped polyneuropathy interval on CFA 13 contained a compelling functional candidate gene, *NDRG1*. This gene is known to be implicated in recessive inherited human Charcot-Marie-Tooth disease type 4D (CMT4D; hereditary motor and sensory neuropathy-Lom) [21]. In addition, *Ndrg1*-deficient mice have shown that NDRG1 is essential for maintenance of the myelin sheaths in peripheral nerves [22]. Histological analysis of the sciatic nerve of *Ndrg1*-deficient mice showed degenerated with demyelination at about five weeks [22]. Although myelination of Schwann cells in the sciatic nerve was normal for two weeks after birth, subsequently, *Ndrg1*-deficient mice showed progressive muscle weakness, especially in the hind limbs. As the age of onset, the clinical course of the disease, and the described nerve pathology in polyneuropathy affected Greyhound show dogs is highly similar to CMT4D affected children and *Ndrg1*-deficient mice, we analyzed the canine *NDRG1* for possible mutations. DNA sequencing revealed a 10 bp deletion in the *NDRG1* gene, which is perfectly associated with the polyneuropathy phenotype in Greyhound show dogs. We confirmed the presence of this mutation on the genomic DNA and mRNA level. Interestingly, we detected a significantly reduced steady-state abundance of *NDRG1* transcript in affected nerves compared to the situation in healthy dogs. Nonsense-mediated decay (NMD) selectively recognizes and degrades mRNAs whose open reading frame is truncated by a premature translation termination codon [23]. As the canine *NDRG1* deletion leads to a different and longer terminal portion of the protein, NMD seems to be unlikely as the causative mechanism for this observation, thus indicating the existence of alternative RNA decay mechanisms.

While it was unclear whether the mutant protein is actually expressed, we speculated that due to the frameshift, any mutant protein produced will contain 61 altered amino acids. Thus, mutant protein might be misfolded and exhibit protein dysfunction, and also could potentially interfere with normal cellular function. A database search for possible identical proteins revealed no results and the Western blot analysis detected no mutant protein in peripheral nerve from a polyneuropathy affected dog, likely due to the absence of the protein. The normal cellular function of the altered NDRG1 may not be maintained. Taken together, we think that the most likely explanation for the polyneuropathy phenotype in affected Greyhound show dogs is lack of NDRG1 caused by the 10 bp deletion. The analysis of NDRG1 expression using an antibody which is predicted to detect the wild-type and the mutant protein in neural tissue extracts of an affected and a healthy control dog confirmed our hypothesis. Obviously, a complete lack of NDRG1 in homozygous mutant dogs causes the severe course of the disease, as previously reported in *Ndrg1*-deficient mice [22].

Our finding of a *NDRG1* mutation in dogs with polyneuropathy provides a valuable model for human medicine possibly useful for the investigation of the pathogenesis and therapeutic trials. The naturally occurring polyneuropathy dog model may represent a better model for human CMT4D than *Ndrg1*-deficient mice because of the dog's body size and structure and the resulting similarity to the human situation. In addition, the longer life expectancy of dog allows for investigations over a longer time period. Dogs have the additional advantages of being economic to maintain and having been bred for easy management. Moreover, a high level of expertise in reproductive technology and veterinary care is available for them.

In conclusion, by positional cloning we have identified the 10 bp deletion in the canine *NDRG1* gene as the most likely causative mutation for polyneuropathy in Greyhound show dogs. This result allows careful eradication of this genetic disease from the worldwide Greyhound breeding population. Our study also provides a defined animal model for similar human hereditary CMT diseases and confirms NDRG1 critical function for myelination of peripheral nerves.

## Materials and Methods

### Animals

We collected samples from polyneuropathy affected Greyhound show dogs (n = 10), their healthy littermates (n = 11), sires (n = 6), dams (n = 6), and grandparents (n = 6). Affected dogs derived from Sweden, Denmark, Latvia, France, and Germany. All samples were submitted from private dog owners for research purpose only in accordance with the institutional ethical guidelines. Parents of affected offspring were classified as obligate carriers (n = 14). We also collected 245 not closely related healthy Greyhound show dogs and 112 Greyhound racing dogs resulting in a total of 395 samples from the Greyhound breed. Furthermore, we sampled 16 unrelated control dogs from seven sighthound breeds, which are greyhound shaped dogs (Afghan Hound (n = 2), Borzoi (n = 1), Galgo Español (n = 1), Italian Greyhound (n = 1), Irish Wolfhound (n = 1), Saluki (n = 1), Whippet (n = 9)) and 75 dogs from 75 different breeds for the genotyping of the *NDRG1* exon 15 deletion.

### Clinical characterization of polyneuropathy

Six affected Greyhound show dogs underwent a complete neurological examination according to standardized protocols by different veterinary neurologists because of weakness and progressive gait abnormalities of insidious onset between three to nine months within the first year of life. Electrophysiological evaluations were performed in 5 affected dogs under varying types of general anaesthesia. An electromyogram was recorded from virtually all muscles of front and hind limbs, paraxial musculature and several muscles of the head. Nerve conduction studies and repetitive nerve stimulation was carried out on ulnar, peroneal, and tibial nerves.

### Histopathological examination of nerve and muscle biopsies

Nerve and muscle biopsies were obtained from four animals. Muscle samples were either snap-frozen in isopentane cooled in liquid nitrogen or immersed in 10% neutral-buffered formalin for paraffin embedding. Cryosections were stained with haematoxylin-eosin (HE), modified Gomori trichrome stain, oil red O, and periodic acid Schiff reaction. Moreover, fibre type differentiation was achieved through ATPase reaction and enzyme histochemistry for reduced NADH-TR. Formalin-fixed muscle samples underwent HE and Goldner's trichrome staining. Obtained nerve samples were prepared under a stereolupe and fixed by 2.5% glutaraldehyde in 0.1 M Soerensen's phosphate buffer. Thereafter, fascicles underwent OsO_4_ impregnation and were either embedded in epoxy resin, or immersed in glycerol for single nerve fibre teasing. Plastic embedded material gave rise to semi- and ultrathin sections, with the latter analysed through transmission electron microscopy in a Zeiss EM 10® at 80 kV and a magnification from ×1,700 to ×80,000.

### DNA and RNA extraction

Genomic DNA was isolated from blood or tissue using the Nucleon Bacc2 kit (GE Healthcare). Total RNA was isolated using Trizol reagent (Invitrogen) and DNaseI (Roche) digested according to the manufacturer's instructions.

### Genome-wide homozygosity mapping of the polyneuropathy mutation

Genomic DNA from 7 cases and 17 related control dogs was genotyped on the canine Affymetrix version 2 single nucleotide polymorphism (SNP) genotyping microarray (49,663 SNPs). The results were analyzed with PLINK [24]. After removing 11,604 SNPs with low genotyping success (failed calls >0.3) the average genotyping rate per individual was 81%. A total of 15,034 out of the remaining 38,059 SNPs were polymorphic. Two small nuclear families segregating for polyneuropathy were included to check SNPs for Mendelian inheritance. To identify extended homozygous regions with allele sharing across cases and controls, the following PLINK options were used: –dog –homozyg-kb 100 –homozyg-gap 2500 –homozyg-density 250 –homozyg-match 0.95 –homozyg-window-snp 20 –homozyg-window-missing 5 –homozyg-group –maf 0 –max-maf 1.0 –geno 0.3 –hwe 0 –mind 0.3. All given positions correspond to the CanFam2.0 genome assembly [25]. The corresponding human chromosome segment was identified by BLASTN searches of canine SNP flanking sequences to the human genome sequence.

### Mutation analysis of canine *NDRG1*

The established canine cDNA sequence was used for genomic sequence alignment with the current CFA 13 sequence to characterize the exact gene structure (Figure 4). Primers for the amplification of each of the 15 *NDRG1* coding exons with flanking intronic regions were designed with the software Primer3 (http://frodo.wi.mit.edu/cgi-bin/primer3/primer3_www.cgi) after masking repetitive sequences with RepeatMasker [26]. Primer sequences and PCR conditions are available on request. For the mutation analysis PCR products were amplified of 2 affected and 2 unrelated healthy dogs using AmpliTaqGold®360 DNA polymerase (Applied Biosystems). The subsequent re-sequencing of the PCR products was performed after rAPid alkaline phosphatase (Roche) and exonuclease I (New England Biolabs) treatment using both PCR primers with the ABI BigDye Terminator Sequencing Kit 3.1 (Applied Biosystems) on an ABI 3730 genetic analyzer. Sequence data were analyzed with Sequencher 4.9 (GeneCodes). Fragment size analyses for the genotyping of the *NDRG1* exon 15 deletion were also performed on an ABI 3730 capillary sequencer and analyzed with the GeneMapper 4.0 software (Applied Biosystems).

### RT-PCR

Aliquots of 500 ng total RNA were reverse transcribed into cDNA using 20 pmol (T)_24_V primer and Superscript II reverse transcriptase (Invitrogen). Two microliters of the cDNA were used as a template in RT-PCR.

### Western blot analysis

For Western blot analysis, peripheral nerve biopsies were homogenized in extraction buffer (20 mM Tris, 2% Triton-×100, 20% 5× Laemmli buffer) and protein concentration was determined by BCA assay. Equal amounts of proteins were loaded per lane on 10% SDS-polyacrylamide minigel. The following primary antibodies were used: rabbit polyclonal antibody against human NDRG1 (Sigma-Aldrich HPA006881, immunogen corresponded with 98% identity to aa163-284 of canine NDRG1), and rabbit monoclonal antibody against ACTB (Cell Signaling Technology).

## Supporting Information

Table S1 *NDRG1* polymorphisms.
(0.06 MB PDF)

Figure S1 Selected electrophysiological registrations from a 9-month-old Greyhound affected by polyneuropathy.
(0.13 MB PDF)

Video S1 Polyneuropathy affected Greyhound show dog (age of 3 month).
(3.08 MB MOV)

Video S2 Polyneuropathy affected Greyhound show dog (age of 9 month).
(9.67 MB MOV)
