# Supplementary material for: A Deletion in the N-Myc Downstream Regulated Gene 1 (NDRG1) Gene in Greyhounds with Polyneuropathy
Source: PLoS One. 2010 Jun 22;5(6):e11258. doi: 10.1371/journal.pone.0011258 (PMC2889825; doi:10.1371/journal.pone.0011258)
Supplement: Table S1 — NDRG1 polymorphisms. (0.06 MB PDF) [file pone.0011258.s001.pdf]

Table S1. *NDRG1* polymorphisms

		exon 1	*intron 1*	*intron 1*	*intron 3*	*intron 7*	*exon 9*	*intron 9*	*intron 14*	*intron 14*	*exon 15*
Boxer assembly		G	A	C	T	G	T	G	A	T	TCGCCTGGAC
		c.51G>A	c.63+37A>T	c.63+41C>T	c.206-68T>C	c.538-42G>A	c.675T>C	c.698+68G>A	c.943+36A>G	c.943+51T>C	c.1080_1089delTCGCCTGGAC
case	GY014	AA	TT	TT	CC	AA	CC	GG	GG	TT	del/del
case	GY028	AA	TT	TT	CC	AA	CC	GG	GG	TT	del/del
carrier	GY022	AA	TT	TT	CC	AA	CC	GG	GG	TT	wt/del
carrier	GY025	AA	TT	TT	CC	AA	CC	GG	GG	TT	wt/del
control	GY033	AA	TT	TT	CC	AA	CC	AG	GG	TT	wt/wt
control	GY038	AA	TT	TT	CC	AA	CC	AG	GG	CT	wt/wt
