# Supplementary material for: A Deletion in the N-Myc Downstream Regulated Gene 1 (NDRG1) Gene in Greyhounds with Polyneuropathy
Source: PLoS One. 2010 Jun 22;5(6):e11258. doi: 10.1371/journal.pone.0011258 (PMC2889825; doi:10.1371/journal.pone.0011258)

Figure S1. Selected electrophysiological registrations from a 9-month-old Greyhound affected by polyneuropathy.
(**A, B, C**) Electromyography from left infraspinatus, short digital extensor and cranial tibial muscles, showing pathological spontaneous activity in the form of positive sharp waves.
(**D**) Motor nerve conduction studies from left ulnar nerve, showing abnormally slow nerve conduction velocity (37.3 m/s) and a decrease in the amplitude of the compound muscle action potential.

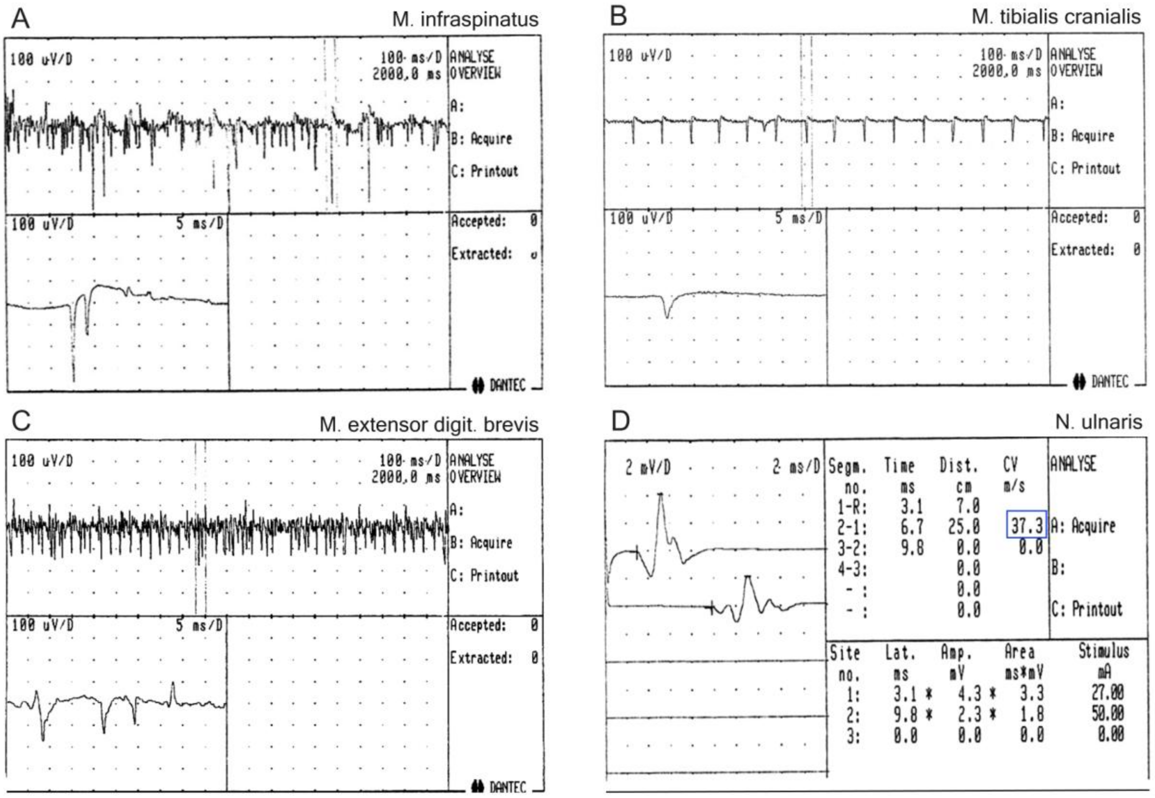

Supplement: Figure S1 — Selected electrophysiological registrations from a 9-month-old Greyhound affected by polyneuropathy. (0.13 MB PDF) [file pone.0011258.s002.pdf]
